# Phosphorylation regulates the sensitivity of voltage‐gated Kv7.2 channels towards phosphatidylinositol‐4,5‐bisphosphate

**DOI:** 10.1113/JP273274

**Published:** 2016-11-07

**Authors:** Isabella Salzer, Fatma Asli Erdem, Wei‐Qiang Chen, Seok Heo, Xaver Koenig, Klaus W. Schicker, Helmut Kubista, Gert Lubec, Stefan Boehm, Jae‐Won Yang

**Affiliations:** ^1^ Department of Neurophysiology and NeuropharmacologyCenter for Physiology and PharmacologyMedical University of Vienna1090ViennaAustria; ^2^ Institute of PharmacologyCenter for Physiology and PharmacologyMedical University of Vienna1090ViennaAustria; ^3^ Department of PediatricsMedical University of Vienna1090ViennaAustria; ^4^ Department of Pharmaceutical ChemistryUniversity of Vienna1090ViennaAustria

**Keywords:** ion channel, KCNQ2, phosphorylation, PIP2

## Abstract

**Key points:**

- Phosphatidylinositol‐4,5‐bisphosphate (PIP_2_) is a key regulator of many membrane proteins, including voltage‐gated Kv7.2 channels.
- In this study, we identified the residues in five phosphorylation sites and their corresponding protein kinases, the former being clustered within one of four putative PIP_2_‐binding domains in Kv7.2.
- Dephosphorylation of these residues reduced the sensitivity of Kv7.2 channels towards PIP_2_.
- Dephosphorylation of Kv7.2 affected channel inhibition via M_1_ muscarinic receptors, but not via bradykinin receptors.
- Our data indicated that phosphorylation of the Kv7.2 channel was necessary to maintain its low affinity for PIP_2_, thereby ensuring the tight regulation of the channel via G protein‐coupled receptors.

**Abstract:**

The function of numerous ion channels is tightly controlled by G protein‐coupled receptors (GPCRs). The underlying signalling mechanisms may involve phosphorylation of channel proteins and participation of phosphatidylinositol‐4,5‐bisphosphate (PIP_2_). Although the roles of both mechanisms have been investigated extensively, thus far only little has been reported on their interaction in channel modulation. GPCRs govern Kv7 channels, the latter playing a major role in the regulation of neuronal excitability by determining the levels of PIP_2_ and through phosphorylation. Using liquid chromatography‐coupled mass spectrometry for Kv7.2 immunoprecipitates of rat brain membranes and transfected cells, we mapped a cluster of five phosphorylation sites in one of the PIP2‐binding domains. To evaluate the effect of phosphorylation on PIP_2_‐mediated Kv7.2 channel regulation, a quintuple alanine mutant of these serines (S427/S436/S438/S446/S455; A^5^ mutant) was generated to mimic the dephosphorylated state. Currents passing through these mutated channels were less sensitive towards PIP_2_ depletion via the voltage‐sensitive phosphatase Dr‐VSP than were wild‐type channels. *In vitro* phosphorylation assays with the purified C‐terminus of Kv7.2 revealed that CDK5, p38 MAPK, CaMKIIα and PKA were able to phosphorylate the five serines. Inhibition of these protein kinases reduced the sensitivity of wild‐type but not mutant Kv7.2 channels towards PIP_2_ depletion via Dr‐VSP. In superior cervical ganglion neurons, the protein kinase inhibitors attenuated Kv7 current regulation via M_1_ receptors, but left unaltered the control by B2 receptors. Our results revealed that the phosphorylation status of serines located within a putative PIP_2_‐binding domain determined the phospholipid sensitivity of Kv7.2 channels and supported GPCR‐mediated channel regulation.

## Key points

- Phosphatidylinositol‐4,5‐bisphosphate (PIP_2_) is a key regulator of many membrane proteins, including voltage‐gated Kv7.2 channels.
- In this study, we identified the residues in five phosphorylation sites and their corresponding protein kinases, the former being clustered within one of four putative PIP_2_‐binding domains in Kv7.2.
- Dephosphorylation of these residues reduced the sensitivity of Kv7.2 channels towards PIP_2_.
- Dephosphorylation of Kv7.2 affected channel inhibition via M_1_ muscarinic receptors, but not via bradykinin receptors.
- Our data indicated that phosphorylation of the Kv7.2 channel was necessary to maintain its low affinity for PIP_2_, thereby ensuring the tight regulation of the channel via G protein‐coupled receptors.

**Abbreviations**
A^5^ mutant: S427/436/438/446/455A mutant
CaM: calmodulin
CaMKIIα: calcium/calmodulin‐dependent protein kinase IIα
CDK5: cyclin dependent kinase 5
CFP: cyan fluorescent protein
CK2: casein kinase 2
D^5^ mutant: S427/436/438/446/455D mutant
DNAPK: DNA‐dependent protein kinase
Dr‐VSP: *Danio rerio* voltage‐sensitive phosphatase
ESI: electrospray ionization
GFP: green fluorescent protein
GPCR: G protein‐coupled receptor
Gq: Gq/11α G protein subunit
GSK3β: gycogen synthase kinase‐3β
GST‐A^5^C
: glutathione S‐transferases‐tagged Kv7.2 C‐terminal ends of the S427/436/438/446/455A mutant
GST‐Kv7.2C: GST‐tagged Kv7.2 C‐terminal ends of wild‐type Kv7.2
IP_3_: inositol 1,4,5 trisphosphate
IPTG: isopropyl β‐D‐1‐thiogalactopyranoside
IT: ion trap
KI‐MIX: four protein kinase inhibitors
LC‐MS/MS: liquid chromatography‐coupled tandem mass spectrometry
mAChR: muscarinic acetylcholine receptor
OxoM: oxotremorine methiodide
p38 MAPK: p38 mitogen‐activated protein kinase
PI(4)P: phosphatidylinositol 4‐phosphate
PI(3,4)P_2_: phosphatidylinositol 3,4‐bisphosphate
PI(4,5)P_2_ or PIP_2_: phosphatidylinositol 4,5‐bisphosphate
PI(3,4,5)P_3_: phosphatidylinositol 3,4,5‐trisphosphate
PKA: cyclic AMP‐dependent protein kinase
PKC: protein kinase C
PLC: phospholipase C
*p*S: phosphoserine
QTOF: quadrupole‐time‐of‐flight
RBM: rat‐brain membrane
SCG: superior cervical ganglion
YFP‐PH: yellow fluorescent protein‐tagged pleckstrin homology domain of PLCδ
wt: wild‐type

## Introduction

Protein phosphorylation of ion channels is vital for neuronal function (Walaas & Greengard, 1991). It has recently become clear that ion‐channel function also relies on the availability of phosphatidylinositol 4,5‐bisphosphate (PIP_2_; Gamper & Shapiro, 2007), one of the most common phosphoinositides in the plasma membrane (Falkenburger *et al*. 2010; Leitner *et al*. 2015). Ion‐channel phosphorylation and PIP_2_ availability are governed by protein kinases and phospholipases, respectively, whose latter activities are controlled by G protein‐coupled receptors (GPCRs). Although the fine tuning of voltage‐gated ion channels via either of these two GPCR‐dependent signalling pathways is well described, so far less attention has been paid to a possible direct link between channel phosphorylation and sensitivity towards PIP_2_ (Liou *et al*. 1999; Lopes *et al*. 2007; Kosenko *et al*. 2012; Shi *et al*. 2014; Zhang *et al*. 2014).

Kv7 potassium channels are tightly controlled by GPCRs (Delmas & Brown, 2005), and neuronal members of the Kv7 family are referred to as Kv7.2 through to 7.5. The most abundant subunit composition in the various types of neurons is a heterotetramer of Kv7.2 and Kv7.3 (Delmas & Brown, 2005). Such heteromers also predominate in sympathetic neurons (Hadley *et al*. 2003), where currents passing through these channels were first described (Brown & Adams, 1980). As these currents can be inhibited through activation of muscarinic acetylcholine receptors (mAChRs), they were termed as M‐currents (Brown & Adams, 1980). In the case of muscarinic M_1_ receptors, depletion of membrane‐bound PIP_2_ is sufficient to fully inhibit currents passing through Kv7 channels (Suh & Hille, 2002). This depletion is effected upon stimulation of a Gq/11α G protein subunit (Gq)‐coupled receptor, whereby activated phospholipase Cβ (PLCβ) hydrolyses PIP_2_ into soluble inositol 1,4,5‐trisphosphate (IP_3_) and membrane‐bound diacylglycerol (Delmas & Brown, 2005). In contrast to mAChRs, bradykinin B2 receptors inhibit M‐currents not via the depletion of membrane PIP_2_, but through an increase in IP_3_ and intracellular Ca^2+^, the latter blocking the channel via calmodulin (CaM) (Cruzblanca *et al*. 1998; Bofill‐Cardona *et al*. 2000; Yus‐Najera *et al*. 2002; Delmas & Brown, 2005).

In addition to the above mechanisms, phosphorylation was also suggested to be involved in Kv7 channel regulation via GPCRs. For instance, protein kinase C (PKC) contributes to M‐current inhibition via M_1_ mAChRs by phosphorylating the Kv7.2 C‐terminus at position serine (S) 541 (Hoshi *et al*. 2003), which leads to CaM dissociation and a resulting decrease in PIP_2_ binding (Kosenko *et al*. 2012). Similarly, src tyrosine kinase causes M‐current suppression by phosphorylating the N‐ and C‐termini of Kv7.3 (Li *et al*. 2004). In contrast, cyclic AMP increases Kv7.1 activity and Kv7.2/7.3 currents through phosphorylation at S27 and S52 in Kv7.1 and Kv7.2, respectively (Schroeder *et al*. 1998; Boucherot *et al*. 2001; Marx *et al*. 2002). A mass spectrometric study proposed phosphorylation sites at T217 in Kv7.2 (and/or equivalently T246 in Kv7.3) and at either S578 or T579 in Kv7.3 (Surti *et al*. 2005). Later on, large‐scale proteome studies identified a number of phosphorylated amino acid residues in Kv7.2 channels, but the implication of different phosphorylation states remains unknown (Trinidad *et al*. 2008, 2012; Tweedie‐Cullen *et al*. 2009; Huttlin *et al*. 2010; Wisniewski *et al*. 2010; Lundby *et al*. 2012). In addition to the Kv7.2 channels themselves, phosphorylation may affect proteins associated with them, such as CaM; phosphorylation of the latter by casein kinase 2 (CK2) enhances M‐currents and reduces their sensitivity towards PIP_2_ depletion (Kang *et al*. 2014).

Here, we aimed to identify constitutive phosphorylation sites in Kv7.2 channels and characterize their contribution to channel function. Using liquid chromatography‐coupled tandem mass spectrometry (LC‐MS/MS), we searched for phosphorylated residues within one of the putative PIP_2_ binding domains. To characterize further these residues both in terms of channel sensitivity towards PIP_2_ and regulation by M_1_ mAChRs, site‐directed mutagenesis was applied. In addition, the cognate protein kinases were identified, and their direct involvement in phosphorylating the residues was investigated using specific inhibitors. We discuss our results with reference to the role of phosphorylation in the PIP_2_ sensitivity of Kv7.2 channels and their regulation by GPCRs.

## Methods

### Ethical approval

Rats were killed by decapitation after CO_2_ asphyxia in accordance with the ARRIVE guidelines and the Austrian animal protection law (http://www.ris.bka.gv.at/Dokumente/BgblAuth/BGBLA_2012_I_114/BGBLA_2012_I_114.pdf) and the Austrian animal experiment by‐laws (http://www.ris.bka.gv.at/Dokumente/BgblAuth/BGBLA_2012_II_522/BGBLA_2012_II_522.pdf) that implement European law (directive 2010/63/EU; see http://eur-lex.europa.eu/LexUriServ/LexUriServ.do?uri=OJ:L:2010:276:0033:0079:en:PDF) into Austrian law.

### Primary cultures of rat superior cervical ganglion neurons

Primary cultures of dissociated superior cervical ganglion (SCG) neurons from newborn rats were prepared as described previously (Salzer *et al*. 2014). Newborn Sprague–Dawley rats were kept and killed 5–8 days after birth by decapitation. Immediately, ganglia were removed, cut into three to four pieces and incubated in collagenase (1.5 mg ml^−1^; Sigma, Vienna, Austria) and dispase (3.0 mg ml^−1^; Sigma) for 30 min at 37°C. Thereafter, ganglia were trypsinized (0.25% trypsin; Worthington, Lakewood, NJ, USA) for 15 min at 37°C, dissociated by trituration and resuspended in Dulbecco's modified Eagle's medium (DMEM; Sigma) containing 4.5 g l^−1^ glucose, 10 mg l^−1^ insulin, 25,000 IU l^−1^ penicillin, 25 mg l^−1^ streptomycin, 50 μg l^−1^ nerve growth factor (R&D Systems, Minneapolis, MN, USA) and 5% fetal bovine serum (FBS; Sigma). For electrophysiological recordings, dissociated cells were seeded onto 35 mm culture dishes coated with rat tail collagen (Biomedical Technologies, Stoughton, MA, USA). The cultures were stored for 4–8 days in a humidified 5% CO_2_ atmosphere at 37°C. On days 1 and 4 after dissociation, the medium was exchanged entirely. For confocal microscopy, freshly dissociated neurons were transfected with yellow fluorescent protein‐tagged pleckstrin homology domain of PLCδ (YFP‐PH) using the Amaxa Basic Neuron SCN Nucleofector Kit (Lonza, Cologne, Germany). Thereafter, cells were seeded onto glass cover slips 1.5H (Marienfeld, Lauda‐Königshofen, Germany) coated with rat tail collagen and stored in a humidified 5% CO_2_ atmosphere at 37ºC for 2 days.

### cDNA constructs

Human Kv7.2 (isoform 4) in pcDNA3 and pECFP‐N1, human Kv7.3 in pcDNA3 (Bal *et al*. 2010) and *Danio rerio* voltage‐sensitive phosphatase (Dr‐VSP) (Hossain *et al*. 2008) in a pIRES2 vector followed by pEGFP were kindly provided by Mark Shapiro (University of Texas Health Science Centre at San Antonio). YFP‐PH (Heo *et al*. 2006) was kindly provided by Tobias Meyer (Department of Chemical and Systems Biology, Stanford University Medical School). Mutagenesis of recombinant human Kv7.2 (KCNQ2)/pcDNA3 was performed using the Quik‐Change site‐directed mutagenesis kit (Stratagene, La Jolla, CA, USA) according to the manufacturer's instructions. The full‐length Kv7.2 C‐terminal ends of the wild‐type (wt) and A^5^ mutant (GST‐Kv7.2C and GST‐A^5^C; residues 317–841) were generated by PCR with 5′ primers carrying a *Bam*HI site and 3′ primers with a *Hind*III site with each Kv7.2 wt and A^5^ in pcDNA3. After a double digest with the restriction enzymes, the PCR product was cloned in‐frame into a pre‐digested pGEX vector to obtain pGEX‐Kv7.2C tagged with a GST and a 7xHis at its C‐ and N‐termini, respectively.

### Cell culture and transient transfection

Cultured tsA201 cells, a transformed HEK 293 cells stably expressing the SV40 large T‐antigen, were propagated in DMEM with 10% FBS. Cells were maintained in a humidified atmosphere of 5% CO_2_ at 37°C and transiently transfected using ExGen500 or Turbofect (Thermo Fisher, Rockford, lL, USA) according to the manufacturer's protocol.

### Immunopurification and immunoblot of Kv7.2

Rat brain membranes (RBMs) and cell lysates from tsA201 cells transiently expressing KCNQ2/pECFP‐N1 were prepared as described previously (Foster *et al*. 2012). RBMs and cell lysates were solubilized in 1% Triton X‐100 lysis buffer and incubated overnight with a rabbit anti‐KCNQ2 (AB22897, Abcam, Cambridge, UK; 1–2 μg ml^−1^) or a mouse anti‐KCNQ2 (N26A/23, NeuroMab, Davis, CA, USA; 1–2 μg ml^−1^), and rabbit anti‐GFP polyclonal antibodies (A6455, Thermo Fisher; 1–2 μl ml^−1^), respectively. Antigen–antibody complexes were then incubated with protein G beads (GE Healthcare, Uppsala, Sweden) for 4 h at 4°C. After six washes, bound proteins were eluted in SDS sample buffer at 95°C for 3 min. Eluted proteins were size fractionated on SDS‐PAGE gels, and visualized by a colloidal blue staining (Thermo Fisher). For immunoblot with GST‐Kv7.2C, purified proteins were separated on SDS‐PAGE gels and transferred to polyvinylidene fluoride membranes (Waters, Milford, MA, USA), which were then immunostained with anti‐KCNQ2 (N26A/23, 0.22 μg ml^−1^) antibody.

### In‐gel digestion

The Kv7.2 bands were directly excised from SDS‐PAGE gels, destained with 50% acetonitrile in 50 mm ammonium bicarbonate, and dried in a speed vacuum concentrator. After reduction and alkylation of Cys, gel pieces were washed and dehydrated. Dried gel pieces were swollen with 25 mm ammonium bicarbonate (pH 8.0) containing 10 ng μl^−1^ trypsin (Promega, Madison, WI, USA) or chymotrypsin (Promega) and incubated at 37°C for 2–18 h. Digested peptides were extracted with 50% acetonitrile in 5% formic acid and concentrated in a speed vacuum concentrator (Yang *et al*. 2007).

### LC‐MS/MS

An electrospray ionization (ESI) ion trap (IT) mass spectrometer (HCT and Amazon speed ETD, Bruker, Bremen, Germany) and an ESI‐quadrupole‐time‐of‐flight (QTOF; Compact, Bruker) coupled with an UltiMate 3000 Nano‐HPLC system (Dionex, Thermo Fisher) were used for LC‐MS/MS data acquisition. A PepMap100 C‐18 trap column (300 μm × 5 mm) and PepMap100 C‐18 analytical column (75 μm × 150 mm) were used for reverse phase (RP) chromatographic separation with a flow rate of 300 nl min^−1^. The two buffers used for the RP chromatography were 0.1% formic acid (FA)/water (buffer A) and 0.08% FA/80% acetonitrile/water (buffer B) with a 125 min gradient (4–30% B for 105 min, 80% B for 5 min and 4% B for 15 min) or a 207 min gradient (4–10% B for 67 min, 20% B for 50 min, 40% B for 60 min, 65% B for 10 min, 99% B for 7 min and 4% B for 13 min). Eluted peptides were then directly sprayed into the mass spectrometer to record peptide spectra over the mass range of *m*/*z* 300–2000 and MS/MS spectra in information‐dependent data acquisition over the mass range of *m*/*z* 50–2800. Repeatedly, MS spectra were recorded followed by three data‐dependent MS/MS spectra generated from the four highest intensity precursor ions. The MS/MS spectra were interpreted with the Mascot search engine (Matrix Science, London, UK) with a mass tolerance of 0.5 Da (IT) or 20 p.p.m. (QTOF), an MS/MS tolerance of 0.5 Da (IT) or 0.1 Da (QTOF); one to three missing cleavage sites and carbamidomethylation on Cys, oxidation on Met and phosphorylation on Ser/Thr were allowed. Each filtered MS/MS spectrum exhibiting possible phosphorylation was manually checked and validated. Existence of a 98 Da mass loss (–H_3_PO_4_: phosphopeptide specific neutral loss) and any ambiguity of phosphorylation sites were carefully examined (Yang *et al*. 2007; Foster *et al*. 2012).

### Purification of GST‐Kv7.2C and *in vitro* protein kinase assay

GST‐Kv7.2C and GST‐A^5^C fusion proteins were expressed in transformed *E. coli* XL1 Blue and BL21 with isopropyl β‐D‐1‐thiogalactopyranoside (IPTG, 0.5 mM) induction. After cell lysis using the French press, GST fusion proteins were pulled down with glutathione sepharose 4 Fast Flow (GE Healthcare) and eluted with glutathione (50 mm, pH 8). Purified GST‐Kv7.2C fusion protein was incubated individually with recombinant cyclin dependent kinase 5 (CDK5)/p35, calcium/calmodulin‐dependent protein kinase IIα (CaMKIIα), p38 mitogen‐activated protein kinase (p38 MAPK), cyclic AMP‐dependent protein kinase (PKA), PKC, glycogen synthase kinase‐3β (GSK3β) and DNA‐dependent protein kinase (DNAPK) (Thermo Fisher). The following protein kinase reaction buffers were used for respective protein kinases: 200 ng of CDK5/p35 in 50 mm Tris (pH 7.6) with 1 mm dithiothreitol (DTT), 1 mm MgCl_2_, 1 mm EGTA and 300 μm ATP; 150 ng of CaMKIIα in 80 mm Mops with 5 mm MgCl_2_, 15 mm CaCl_2_, 2.5 mm calmodulin and 200 μm ATP; 200 ng of p38 MAPK in 50 mm Tris (pH 7.6) with 1 mm DTT, 1 mm MgCl_2_, 1 mm EGTA and 300 μm ATP; 260 ng of PKA in 50 mm Tris (pH 7.6) with 1 mm DTT, 20 mm MgCl_2_, 200 nm cAMP and 300 μm ATP; 200 ng of PKC in 50 mm Tris (pH 7.6) with 1 mm DTT, 10 mm MgCl_2_, 2 mm CaCl_2_ and 300 μm ATP; 160 ng of GSK3β in 50 mm Tris (pH 7.6) with 1 mm DTT, 20 mm MgCl_2_, 300 μm ATP; 200 ng of DNAPK in 50 mm Tris (pH 7.6) with 1 mm DTT, 10 mm MgCl_2_, 10 mm EDTA, 60 mm NaCl, 1 μg activator (provided by the manufacturer) and 300 μm ATP. All reactions were carried out at 30°C for 30 min in a final volume of 50 μl and stopped by adding reducing SDS sample buffer. Proteins were separated by SDS‐PAGE for further in‐gel digestion and LC‐MS/MS.

### *In vitro* phosphorylation assay with [γ‐^32^P]ATP

GST‐Kv7.2C and GST‐A^5^C were phosphorylated *in vitro* with a mixture of recombinant CDK5, p38 MAPK, PKA and CaMKIIα kinases in the presence or absence of four protein kinase inhibitors (1 μm roscovitine, 3 μm SB203580, 10 μm KN‐62 and 10 μm H7; KI‐MIX). Each reaction was performed with 7.5 μg GST‐fusion protein in kinase buffer (50 mm Tris pH 7.6, 6.25 mm DTT, 47 mm MgCl_2_, 33 mm CaCl_2_, 8 mm CaM, 625 μm cAMP, 93.5 ng CaMKIIα, 100 ng CDK5, 144 ng p38 MAPK and 101.75 ng PKA) with solvent or KI‐MIX. A mixture of hot ([γ‐^32^P]ATP, Hartmann Analytics, Braunschweig, Germany) and cold ATP was added at a final ratio of 1:3, resulting in 3.3 pmol of total ATP in each reaction. All steps were carried out on ice until ATP was added, and the tubes were incubated at 30°C for 30 min. Subsequently, the reactions were filled with 50 mm Tris buffer (pH 7.6) and 3 μg of anti‐Kv7.2 antibody (NeuroMab) added for immunoprecipitation. Extracts were used to count the radioactivity in a Packard 1900CA Tri‐Carb liquid scintillation analyser (PerkinElmer, Waltham, MA, USA) and for autoradiography on a 12.5% PhastGel (GE Healthcare).

### Electrophysiology

Recordings of currents through Kv7 channels were performed at room temperature (20–24°C) using the perforated patch‐clamp technique, which prevents their rundown (Salzer *et al*. 2014). Patch pipettes were fabricated from borosilicate glass capillaries (GB150‐8P, Science Products, Hofheim, Germany) using a Sutter P97 puller (Sutter Instruments, Novato, CA, USA) and front filled with a solution containing (in mm): potassium gluconate 133; NaCl 5.9; CaCl_2_ 1.0; MgCl_2_ 0.7; Hepes 10; EGTA 10; KOH 29.4 (pH 7.4). Thereafter, electrodes were backfilled with the same solution including 200 μg ml^−1^ amphotericin B (in 0.8% DMSO), which yielded tip resistances of 0.8–1.5 MΩ. Currents were recorded after 20–30 min when series resistance stabilized below 12 MΩ. The external solution contained (in mm): NaCl 140; glucose 20; Hepes 10; CaCl_2_ 2.5; MgCl_2_ 2.0; KOH 3; NaOH 2 (pH 7.4). The solutions result in a liquid junction potential of 15.6 mV, which was corrected for. For recording ‘sensing currents’ of Dr‐VSP, the external solution contained (in mm): TEA‐Cl 140; glucose 20; Hepes 10; CaCl_2_ 3.0; MgCl_2_ 2.0; XE991 0.003; pH 7.4 was adjusted with TEA‐OH. For recordings involving SCG neurons, tetrodotoxin (0.5 μm; Latoxan, Valence, France) was included in the external solution to prevent currents through voltage‐gated Na^+^ channels. External solutions were applied using a DAD‐12 drug application device (Adams & List, Westbury, NY, USA), permitting a complete solution exchange around the patched cell within <100 ms. To investigate PIP_2_‐mediated changes of currents through heterologously expressed Kv7.3 wt and mutant Kv7.2 channels, co‐transfected Dr‐VSP was activated at +100 mV for (in ms): 10; 20; 40; 80; 160; 320; 640; 1200; 2400; 4800. Baseline Kv7 current levels were determined at −30 mV before Dr‐VSP activation and compared to current levels at −30 mV immediately after Dr‐VSP activation; then currents were allowed to recover fully for 45 s followed by a 5 s step to −80 mV. The obtained data were fitted with a mono‐exponential decay curve. The differences of the fits were calculated with an extra sum of squares *F* test comparing the τ values of the deactivation. Subsequently, ‘sensing currents’, mediated by activation and deactivation of Dr‐VSP, were tested by current–voltage relationships ranging from −60 mV to +160 mV in 10 mV increments and a holding potential of −60 mV. These voltage steps lasted for 100 ms and were evoked once every 2 s. A p/6 protocol was used for leak subtraction with a subpulse holding potential of −100 mV. The traces were lowpass filtered at 10 kHz and sampled at 50 kHz.

To investigate currents through native Kv7 channels in SCG neurons, cells were clamped to −30 mV and hyperpolarized to −55 mV every 15 s. The difference between the tail current amplitude 20 ms after the onset of the pulse to −55 mV and 20 ms prior to repolarization was used to determine the M‐current amplitude (*I*_M_). After a control recording of 120 s, nine concentrations of oxotremorine methiodide (OxoM) (in μm: 0.001; 0.003; 0.01; 0.03; 0.1; 0.3; 1; 3; 10; Sigma) were applied sequentially, each for 120 s, followed by 300 s wash‐out and application of Kv7 channel specific blocker XE991 (3 μm) for 180 s to verify the recording of M‐currents. M‐current amplitudes at the end of the 120 s application of each OxoM concentration were normalized to control levels before application and displayed as percentage *I*_M_ inhibition. The obtained values were fitted with a Hill equation. Significant differences of the fits were calculated using an extra sum of squares *F* test comparing the fitted maxima of each curve. When needed, transfected tsA201 cells and SCG neurons were treated overnight with solvent or KI‐MIX.

### Confocal microscopy

SCG neurons, transfected with a YFP‐tagged PIP_2_ reporter (YFP‐PH), were imaged on a Nikon A1R+ confocal laser scanning microscope (Nikon, Vienna, Austria) using a ×40 1.25 NA water immersion objective with the confocal pinhole set to 1 Airy unit, resulting in a 0.66 μm optical section. Scanning was performed with a 8 kHz resonant scanner unit using pixel dwell times on 100 ns using four times averaging and an *x*/*y* pixel size of 0.044 μm. YFP fluorescence was excited using the 514 nm line of an argon laser at 1.5% maximal intensity, filtered by a dielectric filter (540 nm/30 nm) and detected using a GaAsP photomultiplier tube. Images were taken at a frequency of 2 Hz. Substances were applied using a focal perfusion device (ALA Scientific, Farmingdale, NY, USA).

### Statistics

Statistical significances were calculated using Prism 6 (GraphPad, La Jolla, CA, USA). Data are presented as means ± SEM. Time constants (τ values) are presented as mean values followed by the 95% confidence interval. In electrophysiological recordings and confocal images *n* represents the number of single cells measured. In experiments involving determination of radioactivity, *n* represents the number of independent experiments. Statistical tests are indicated in the figure legends.

## Results

### Identification of *in vivo* phosphorylation sites in Kv7.2

**Figure 1:**
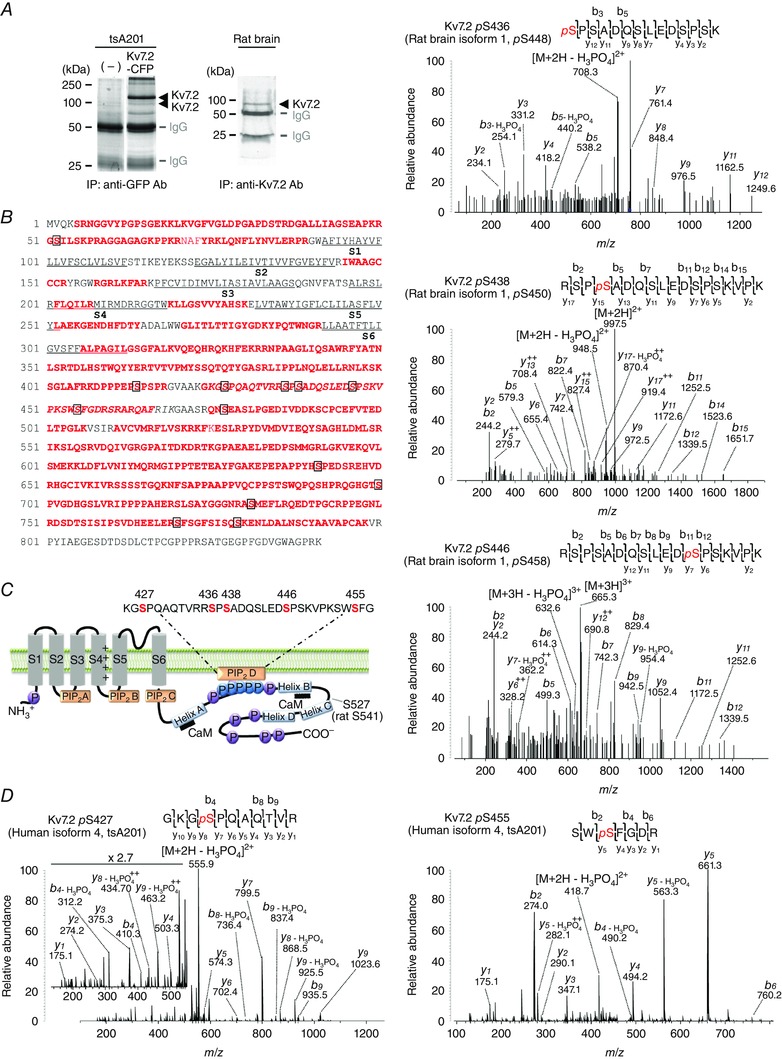
Identification of phosphorylation sites in Kv7.2 by LC‐MS/MS *A*, colloidal blue‐stained SDS‐PAGE gel images with proteins immunopurified by anti‐GFP and anti‐KCNQ2 antibodies (Ab) from tsA201 cells heterologously expressing human Kv7.2 and rat brain, respectively. (–) represents immunopurification with untransfected tsA201 cells via an anti‐GFP Ab, and arrowheads indicate bands containing Kv7.2 protein identified by LC‐MS/MS. *B*, sequence coverage of human Kv7.2 (isoform 4) with identified peptides (bold) by MS/MS from transfected tsA201 cells. The putative transmembrane segments (S1–S6) of Kv7.2 are underlined and the identified phosphorylation sites are indicated in boxes. *C*, membrane topology of Kv7.2 with localization of the identified phosphorylation sites. Note the cluster of phosphorylation (P) sites in one of the four putative PIP_2_ binding regions (PIP_2_A–PIP_2_D). *D*, representative MS/MS spectra for five phosphosites in the PIP_2_D domain of Kv7.2 either heterologously expressed in tsA201 cells or derived from rat brain. The MS/MS spectra of Kv7.2 peptides obtained at *m/z* 604.93, *m/z* 757.30, *m/z* 997.50, *m/z* 665.31 and *m/z* 467.80 present phosphorylated serines (*p*S) at residue 427, 436, 438, 446 and 455, respectively, with β‐eliminated *b‐* and *y‐*ions and neutral loss of H_3_PO_4_ from the precursor. *p*S residue numbers correspond to the human Kv7.2 isoform 4. [Colour figure can be viewed at wileyonlinelibrary.com]

Phosphorylated amino acid residues of Kv7 proteins have been detected previously following specific phosphopeptide enrichment in protein preparations from rodent tissues, including the brain (Trinidad *et al*. 2008, 2012; Tweedie‐Cullen *et al*. 2009; Huttlin *et al*. 2010; Wisniewski *et al*. 2010; Lundby *et al*. 2012). To expose the phosphorylation sites located specifically in mammalian Kv7.2, they were determined in human Kv7.2 (isoform 4 carrying a CFP‐tag) expressed in tsA201 cells as well as in rat Kv7.2 derived from brain preparations. Proteins were immunopurified using anti‐GFP and anti‐KCNQ2 antibodies, respectively (Fig. 1 *A*). After size‐fractionation with SDS‐PAGE (Fig. 1 *A*), the excised colloidal blue‐stained bands were digested in‐gel with trypsin or chymotrypsin and the resulting peptides were subjected to LC‐MS/MS.

The results showed that our method could unambiguously identify Kv7.2 channels (Uniprot ID, O43526‐4; www.uniprot.org/uniprot/O43526) with 74.91% sequence coverage (Fig. 1 *B*). Cytosolic N‐ and C‐termini were covered by 93.41% and 88.66%, respectively. These LC‐MS/MS analyses revealed 13 phosphorylation sites for human Kv7.2 via a Mascot database search followed by manual validation based on β‐eliminated *b*‐ and *y*‐fragment ions and a neutral loss of phosphoric acid (Fig. 1 *D* and Table 1). One phosphoserine (*p*S) at residue 52 was located at the N‐terminus, whereas the remaining phosphoserines were mapped to the C‐terminus (Fig. 1 *B* and *C*). Six of these 13 phosphoserines were also detected in LC‐MS/MS analyses of Kv7.2 channels that were immunopurified from rat brain (Table 1 and Fig. 1 *D*). The 12 C‐terminal phosphorylation sites were divided into two clusters: a proximal cluster containing seven phosphorylated serines in the region from S414 to S477, and a distal cluster of five phosphorylated residues that was more loosely distributed in a stretch extending from S641 to S779 (Fig. 1 *B*). The topographical localization of these phosphoresidues in Kv7.2 is presented in Fig. 1 *C*.

**Table 1:** Phosphorylated sites in Kv7.2 identified by MS

Kv7.2	Sequence	Identified in	Previously identified
pS52	RG***pS***ILSKPR	tsA^a^, rat brain	(Wisniewski *et al*. 2010; Trinidad *et al*. 2012)
pS414	KDPPPEP***pS***PSPR	tsA	(Trinidad *et al*. 2012)
**pS427** b	GKG***pS***PQAQTVR	tsA	(Wisniewski *et al*. 2010)
**pS436**	R***pS***PSADQSLEDSPSK	tsA, rat brain	(Huttlin *et al*. 2010; Wisniewski *et al*. 2010; Trinidad *et al*. 2012)
**pS438**	RSP***pS***ADQSLEDSPSK	tsA, rat brain	(Huttlin *et al*. 2010; Wisniewski *et al*. 2010; Lundby *et al*. 2012)
**pS446**	RSPSADQSLED***pS***PSK	tsA, rat brain	(Wisniewski *et al*. 2010; Trinidad *et al*. 2012)
**pS455**	SW***pS***FGDR	tsA	(Tweedie‐Cullen *et al*. 2009; Huttlin *et al*. 2010; Lundby *et al*. 2012; Trinidad *et al*. 2012)
pS477	QN***pS***EASLPGEDIVDDK	tsA	(Wisniewski *et al*. 2010; Lundby *et al*. 2012; Trinidad *et al*. 2012)
pS641	EPEPAPPYH***pS***PEDSR	tsA, rat brain	(Huttlin *et al*. 2010; Wisniewski *et al*. 2010; Lundby *et al*. 2012; Trinidad *et al*. 2012)
pS700	QGHGT***pS***PVGDHGSLVR	tsA, rat brain	(Huttlin *et al*. 2010; Wisniewski *et al*. 2010; Trinidad *et al*. 2012)
pS731	SLSAYGGGNRA***pS***MEFLR	tsA	—
pS772	SF***pS***GFSISQSK	tsA	(Wisniewski *et al*. 2010)
pS779	SISQ***pS***KENLDALNSCY	tsA	(Trinidad *et al*. 2012)

^a^tsA, tsA201 cells heterologously expressing Kv7.2. ^b^Phosphorylation sites within the PIP_2_D domain (Fig. 1 *C*) are in bold.

Previously, this ion channel has been proposed to contain at least four PIP_2_‐binding domains: two PIP_2_‐binding sites are thought to be located in the S2–S3 and S4–S5 linker regions, involving residues lysine(K)162 and K230, respectively (Zhang *et al*. 2013). A third putative binding region, including residue histidine (H)327, is found in the C‐terminus immediately adjacent to the S6 domain (Telezhkin *et al*. 2013). The fourth presumed PIP_2_‐binding region is located in the C‐terminus, between helices A and B, and harbours a cluster of basic amino acid residues, i.e. residues K425–K469 (Hernandez *et al*. 2008). These regions were indicated as PIP_2_A–PIP_2_D (Fig. 1 *C*).

Hence, it was conspicuous that the phosphoresidues *p*S427, *p*S436, *p*S438, *p*S446, and *p*S455 were clustered accurately within PIP_2_D, which prompted us to investigate their potential role in the PIP_2_ sensitivity of Kv7.2 channels.

### PIP_2_ sensitivity is decreased in the dephosphomimetic mutant of Kv7.2

**Figure 2:**
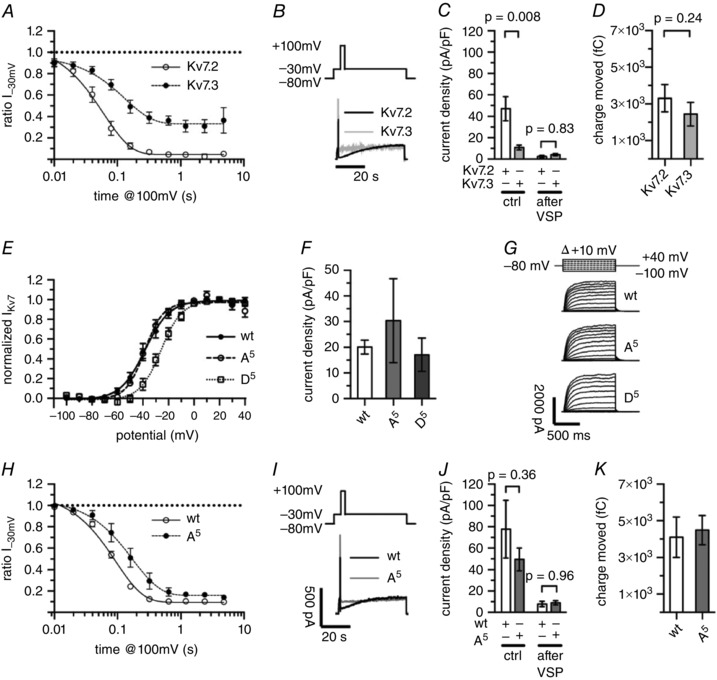
Phosphorylation in a putative PIP_2_ binding motif of Kv7.2 affects PIP_2_ sensitivity *A* and *H*, difference of current ratios at −30 mV of transfected Kv7.2 (*n* = 5) and Kv7.3 (*n* = 5) channels before and after Dr‐VSP activation, *P* = 0.0002 (extra sum‐of‐squares *F* test) (*A*), or Kv7.2 wt (*n* = 9) and Kv7.2 A^5^ mutant (*n* = 8) channels before and after Dr‐VSP activation, *P* < 0.0001 (extra sum‐of‐squares *F* test) (*H*). Dr‐VSP was activated at +100 mV for increasing periods of time (10–4800 ms). *B* and *I*, representative current traces of homomeric Kv7.2 and Kv7.3 (*B*) or Kv7.2 wt and dephosphomimetic Kv7.2 A^5^ mutant channels (*I*) co‐expressed with Dr‐VSP. Note that in *B* currents were normalized in order to exemplify different residual current levels after 160 ms of Dr‐VSP activation at +100 mV. *C* and *J*, current densities were calculated from the data shown in *A* and *H* obtained before (control) and after 640 ms of VSP activation (after VSP). *P* values as indicated (one‐way ANOVA, Holm–Sidak multiple comparisons test). *D* and *K* compare the off‐gating charges mediated by the deactivation of Dr‐VSP from +140 mV to −60mV. Whenever possible, current–voltage (*I–V*) curves from −60 mV to +160 mV in the presence of 145 mm TEA‐Cl and 3 μm XE991 were recorded in the same cells as used for Kv7.2: *n* = 5 and Kv7.3: *n* = 4 (*A*); and Kv7.2 wt: *n* = 6 and Kv7.2 A^5^: *n* = 6 (*H*) (Mann–Whitney test). *E*, activation curves of Kv7.2 wt, A^5^ or D^5^ mutant channels transiently expressed in tsA201 cells. Peak deactivation tail at −80 mV was referred to completely deactivated values. Values were normalized to deactivation tail amplitude of +20 mV. *P* < 0.0001 (extra sum‐of‐squares *F* test; *n* = 6). *F*, current densities of wt and mutant Kv7.2 channels taken from *E* at deactivation tail amplitude of +20 mV, *P* = 0.80 (Kruskal–Wallis test, Dunn's multiple comparisons test). *G*, representative currents recorded in tsA201 cells heterologously expressing Kv7.2 wt, A^5^ or D^5^ mutant channels by the voltage protocol indicated above the current traces.

PIP_2_ is generally required for Kv7 channel opening, and Kv7.2 is particularly sensitive to changes in membrane PIP_2_ levels (Suh *et al*. 2006; Gamper & Shapiro, 2007). To test Kv7.2 for its PIP_2_ sensitivity, channels were coexpressed with the *Danio rerio* voltage‐sensitive phosphatase (Dr‐VSP) that, upon depolarization, predominantly removes the phosphate at position 5 of the inositol ring of PI(4,5)P_2_ (Iwasaki *et al*. 2008; Keum *et al*. 2016) thereby reducing currents through Kv7.2 (Zhang *et al*. 2013; Rjasanow *et al*. 2015). Currents through Kv7 were elicited by switching the membrane potential from −80 to −30 mV, and Dr‐VSP was activated by intermittent depolarizations to +100 mV for 0.01–4.8 s. The ratio of current amplitudes at −30 mV just before and right after these intermittent depolarizations was determined (Fig. 2 *A* and *B*). Initially, currents through homomeric Kv7.2 and Kv7.3 channels were compared, as these two Kv7 channel subunits differ strongly in their PIP_2_ sensitivities. Apparent affinities of Kv7.3 channels for the water‐soluble analogue of PIP_2_ (diC8‐PIP_2_) are in the low micromolar range, whereas those of Kv7.2 are rather in the submillimolar range (Li *et al*. 2005; Telezhkin *et al*. 2012).

The results showed that currents passing through both of these homomers were reduced by Dr‐VSP activation in a time‐dependent manner. However, Kv7.2 was much more sensitive towards PIP_2_ depletion via Dr‐VSP than Kv7.3 (Fig. 2 *A* and *B*). The time constant for deactivation (τ_decay_) of currents through Kv7.2 was 0.061 s (0.050–0.078 s) *versus* 0.134 s (0.080–0.213 s) for currents through Kv7.3. Hence, the higher the apparent affinity of a channel for PIP_2_, the less pronounced are the effects of PIP_2_ depletion (Gamper & Shapiro, 2007).

To reveal whether phosphorylation of the serines within PIP_2_D of Kv7.2 (S427, S436, S438, S446 and S455) may affect the channel's sensitivity towards PIP_2_ depletion, these amino acids were mutated to either alanines (A) to generate the dephosphomimetic mutant S427/436/438/446/455A (A^5^ mutant), or aspartic acid (D), to obtain the phosphomimetic mutant S427/436/438/446/455D (D^5^ mutant). Since such substitutions may change the voltage dependence of K^+^ channels (Park *et al*. 2006), current–voltage relations were obtained for these two Kv7.2 variants.

The results showed that the channel–voltage dependence of the A^5^ mutant was not different from those of wt Kv7.2 channels, as was the current density (Fig. 2 *E* and *F*), whereas the activation curve for the D^5^ mutant was right**‐**shifted by 12 mV (Fig. 2 *E*). To avoid any influence of the altered voltage sensitivity, the D^5^ mutant was not compared with wt Kv7.2. However, when the A^5^ mutant (τ_decay_ = 0.176 s; 0.138–0.244 s) was compared with wt Kv7.2 (τ_decay_ = 0.095 s; 0.085–0.106 s), it was significantly less sensitive towards PIP_2_ depletion via Dr‐VSP (*P* < 0.0001, extra sum‐of‐squares *F* test) (Fig. 2 *H* and *I*).

Thus, phosphorylation of the serines in the PIP_2_D domain of Kv7.2 might play a crucial role in determining its PIP_2_ sensitivity, which could be tested for by locating the cognate protein kinases.

### Identification of protein kinases phosphorylating the PIP_2_ binding motif in Kv7.2

**Figure 3:**
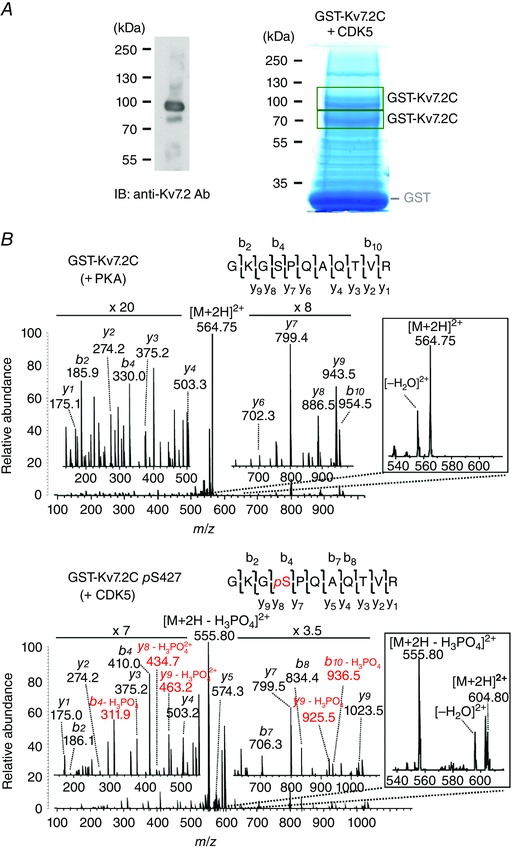
Identification of protein kinases for phosphorylation in the PIP_2_D domain by *in vitro* protein kinase assay and MS analysis *A*, purified GST‐Kv7.2C proteins were visualized by immunoblotting with an anti‐Kv7.2 antibody (left) and colloidal blue staining (right). Gel bands where GST‐Kv7.2C was identified by MS analysis are indicated in boxes (right). *B*, the Kv7.2 S427 was phosphorylated by CDK5, but not by PKA. Representative MS/MS spectra of non‐phosphorylated (top) and phosphorylated (bottom) S427 obtained by *in vitro* phosphorylation assays with the recombinant PKA and CDK5, respectively. The MS/MS spectra were acquired from precursor ions at *m/z* 564.75 for PKA and *m/z* 604.80 for CDK5. The phosphorylation site at S427 was verified by β‐eliminated *b*‐ and *y*‐ions and neutral loss of H_3_PO_4_ from the precursor, a typical signature of phosphopeptides. [Colour figure can be viewed at wileyonlinelibrary.com]

To be able to explore further the functional role of the phosphoserines in the PIP_2_D domain of Kv7.2, it was necessary to identify the protein kinases responsible for phosphorylation. This was elucidated by incubating the purified, GST‐tagged C‐terminal end of Kv7.2 (GST‐Kv7.2C) with each of seven recombinant protein kinases, including CDK5, p38 MAPK, GSK3β and PKA, as predicted in Table 2 by the computer algorithm NetPhosK (Blom *et al*. 2004). After *in vitro* protein kinase reactions, proteins were separated by SDS‐PAGE (Fig. 3 *A*) and residues phosphorylated by specific protein kinases were identified by LC‐MS/MS.

**Table 2:** Identification of protein kinases involved in phosphorylation of Kv7.2 PIP_2_D domain by *in vitro* protein kinase assay and LC‐MS/MS

		*In vitro* protein kinase assays						
Residue	Predicted protein kinase^a^	CDK5	p38	CaMKIIα	PKA	PKCα	GSK3β	DNAPK
pS427	PKA, p38MAPK	+	+	−	−	−	−	−
pS436	p38MAPK, GSK3	−	+	−	−	−	−	−
pS438	PKA	−	−	+	+	−	−	−
pS446	CDK5, cdc2, GSK3	+	+	−	−	−	−	−
pS455	n.p.	−	−	−	+	−	−	−

+, identified by MS/MS; −, no identification. ^a^Protein kinase prediction by NetPhosK (Blom *et al*. 2004). n.p., not predicted.

The results of MS/MS revealed that CDK5, p38 MAPK, CaMKIIα and PKA phosphorylated each one, two or even three of the five serines (S427, S436, S438, S446 and S455) located within the PIP_2_D domain (Table 2, Fig. 3 *B*). When protein kinases were omitted from the incubation, MS could not detect any phosphorylation sites in GST‐Kv7.2C.

Therefore, CDK5, p38 MAPK, CaMKIIα and PKA, but not PKCα, GSK3β or DNAPK, turned out to be able to phosphorylate the serine residues located in PIP_2_D (Table 2), opening the way for direct examination of the consequences to the sensitivity of Kv7.2 towards PIP_2_ by the selective curtailment of phosphorylation activities.

### Protein kinase inhibition reduces Kv7.2 phosphorylation and decreases its sensitivity towards PIP_2_

Based on the protein kinases identified above, we chose the following inhibitors to reduce phosphorylation within PIP_2_D: roscovitine (1 μm), SB203580 (3 μm), KN‐62 (10 μm) and H7 (10 μm), which inhibit CDK5, p38 MAPK, CaMKIIα and PKA, respectively. Phosphorylation levels were determined by the incorporation of [γ‐^32^P]ATP, based on liquid scintillation counting and autoradiography.

**Figure 4:**
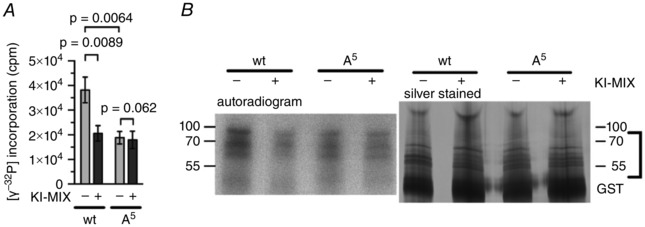
Protein kinase inhibition reduces phosphorylation of the serines in the PIP_2_D domain of Kv7.2 *A* and *B*, GST‐tagged C‐termini of wt (GST‐Kv7.2C, *n* = 5) and A^5^ mutant (GST‐A^5^C, *n* = 5) channels were phosphorylated *in vitro* by the mixture of CDK5, CaMKIIα, p38 MAPK and PKA in the presence (+) or absence (−) of the KI‐MIX. Incorporation of [γ‐^32^P] was determined by liquid scintillation counting (*A*) and autoradiography (*B*, left panel). Background radioactivity in the absence of protein kinases was 844.22 ± 176.23 cpm (mean ± SEM) and 749.50 ± 175.67 cpm for wt and A^5^, respectively. Background radioactivity in the presence of KI‐MIX without protein kinases was 1002.07 ± 87.04 cpm for wt and 833.27 ± 110.73 cpm for A^5^. Comparable loading of purified proteins is shown by silver staining and GST‐Kv7.2C proteins are indicated with brackets based on the identification by LC‐MS/MS (*B*, right panel). *P* values as indicated (one‐way ANOVA, Tukey's *post hoc* test).

The results showed that when wt GST‐Kv7.2C was incubated with the recombinant protein kinases *in vitro* in the presence of a mixture of these inhibitors (KI‐MIX), its phosphorylation was diminished compared to the situation without KI‐MIX (Fig. 4 *A* and *B*). In addition, the results also showed that the dephosphomimetic mutant of GST‐7.2C (GST‐A^5^C) was not phosphorylated to a greater extent than that seen with the diminished phosphorylation of wt GST‐Kv7.2C in the presence of KI‐MIX, and addition of protein kinase inhibitors did not have any further effects (Fig. 4 *A* and *B*).

**Figure 5:**
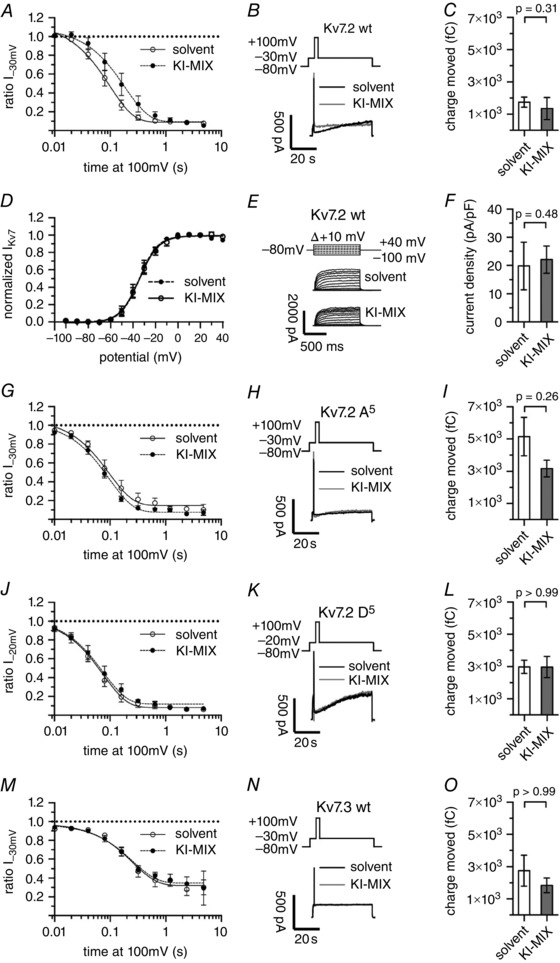
Suppression of phosphorylation affects PIP_2_ sensitivity of Kv7.2 wt channels *A*, *G*, *J* and *M* show changes of relative current levels of protein kinase inhibitor mix (KI‐MIX)‐ and solvent‐treated Kv7.2 wt (*n* = 6; *P* = 0.0008) (*A*), Kv7.2 A^5^ (*n* = 6; *P* = 0.30) (*G*), Kv7.2 D^5^ (*n* = 5; *P* = 0.16) (*J*) and Kv7.3 wt (*n* = 6; *P* = 0.74) (*M*) channels at −30 mV (for Kv7.2 wt, A^5^ and Kv7.3) or −20 mV (for D^5^) before and after activation of Dr‐VSP at +100 mV for different periods of time (10–4800 ms) (extra sum‐of‐squares *F* test). *B*, *H*, *K* and *N*, representative current traces of KI‐MIX‐ and solvent‐treated Kv7.2 wt (*B*), Kv7.2 A^5^ (*H*), Kv7.2 D^5^ (*K*) and Kv7.3 wt (*N*) channels show current reduction after 160 ms of Dr‐VSP activation. *C*, *I*, *L* and *O*, whenever possible, off‐sensing charges of Dr‐VSP were compared in the same cells used for *A*, *G*, *J* and *M*. Off‐sensing values from +140 mV were taken from an *I–V* curve recorded from −60 mV to +160 mV; Kv7.2 wt: *n* = 5 (*C*); Kv7.2 A^5^ solvent‐treated: *n* = 3, A^5^ KI‐MIX‐treated: *n* = 6 (*I*); Kv7.2 D^5^ solvent‐treated: *n* = 4, D^5^ KI‐MIX‐treated: *n* = 3 (*L*); Kv7.3 solvent‐treated: *n* = 4, Kv7.3 KI‐MIX‐treated: *n* = 4 (*O*) (Mann–Whitney test). *D*, activation curves of Kv7.2 channels transiently transfected in tsA201 cells, treated with solvent or KI‐MIX for 12 h (*n* = 6). Peak deactivation tail current at −80 mV was referred to completely deactivated values at −80 mV. Amplitudes of tail currents were normalized to deactivation tail current amplitude for +20 mV, *P* = 0.36 (extra sum‐of‐squares *F* test). *E*, shows representative current traces recorded from tsA201 cells heterologously expressing Kv7.2 wt channels treated with solvent or KI‐MIX for 12 h. The voltage protocol used is indicated above. *F*, current densities of solvent‐ or KI‐MIX‐treated Kv7.2 channels taken from *D* at deactivation tail amplitude of +20 mV, *P* = 0.48 (Mann–Whitney test).

Incubation overnight of tsA201 cells transiently expressing wt Kv7.2 channels in the KI‐MIX rendered the currents through these channels less sensitive towards PIP_2_ depletion via Dr‐VSP (solvent: τ_decay_ = 0.098 s; 0.082–0.121 s, *versus* KI‐MIX: τ_decay_ = 0.184 s; 0.140–0.269 s; Fig. 5 *A* and *B*). However, channel voltage dependence, as determined by current–voltage curves, was not affected by KI‐MIX (Fig. 5 *D–F*). To rule out non‐specific effects due to treatment with KI‐MIX, tsA201 cells expressing the Kv7.2 A^5^ mutant were also incubated in KI‐MIX overnight. The sensitivity of the currents through mutant channels towards PIP_2_ depletion via Dr‐VSP remained unaltered by KI‐MIX (A^5^ solvent: τ_decay_ = 0.107 s; 0.082–0.156 s, *versus* A^5^ KI‐MIX: τ_decay_ = 0.090 s; 0.078–0.106 s, extra sum‐of‐squares *F* test) (Fig. 5 *G* and *H*). Similarly, overnight KI‐MIX incubation of Kv7.2 D^5^ mutant channels did not change the time constant for Dr‐VSP‐mediated current inhibition (D^5^ solvent: τ_decay_ = 0.067 s; 0.054–0.08 s, *versus* D^5^ KI‐MIX: τ_decay_ = 0.088 s; 0.067–0.13 s, extra‐sum‐of‐squares *F* test, Fig. 5 *J* and *K*). However, currents through Kv7.2 D^5^ mutant channels were recorded at −20 mV to compensate for the negative shift in the activation curve (Fig. 2 *E*). In addition, KI‐MIX treatment of cells expressing homomeric wt Kv7.3 channels did not alter the time constant for current reduction (Kv7.3 solvent: τ_decay_ = 0.264 s; 0.192–0.417 s, *versus* Kv7.3 KI‐MIX: τ_decay_ = 0.242 s; 0.184–0.354 s, extra sum‐of‐squares *F* test) by Dr‐VSP activation (Fig. 5 *M* and *N*).

Hence, using recombinant proteins, the effects of the protein kinase inhibitors on the PIP_2_ sensitivity appeared to be specifically related to the serine residues contained within PIP_2_D of Kv7.2. This raised the important issue of whether the same effects could be recapitulated in a native system.

### Phosphorylation of Kv7.2 affects M_1_ receptor‐mediated Kv7 channel inhibition

In sympathetic neurons, the inhibition of M‐currents carried by Kv7 channels via M_1_ mAChRs is mediated by PIP_2_ depletion (Delmas & Brown, 2005). To reveal whether phosphorylation via the protein kinases identified above may also regulate the PIP_2_ sensitivity of neuronal Kv7 channels in a native environment, SCG neurons were incubated overnight in the KI‐MIX. Thereafter, concentration–response curves for the inhibition of M‐currents by the muscarinic agonist OxoM were obtained in such treated neurons and compared with analogous curves derived from neurons treated with solvent instead.

**Figure 6:**
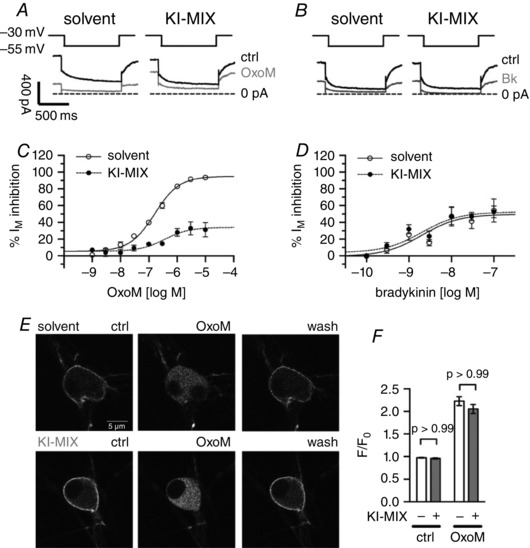
Phosphorylation status of Kv7.2 selectively regulates GPCR‐mediated M‐current inhibition in rat SCG neurons *A* and *B*, current traces show responses of primary cultures of rat SCG neurons, during a 1 s pulse from −30 mV to −55 mV, before (ctrl) and during application of 10 μm OxoM (*A*) and 100 nm of bradykinin (Bk; *B*). Cells were either treated with solvent or KI‐MIX for 12 h. *C* and *D*, concentration–response relations for OxoM‐induced (*C*) or bradykinin‐induced (*D*) M‐current (*I*_M_) inhibition in KI‐MIX‐ and solvent‐treated SCG neurons. *C*, *n* = 5, *P* < 0.0001. *D*, *n* = 5, *P* = 0.36 (extra sum‐of‐squares *F* test). *E*, representative confocal images of SCG neurons transfected with YFP‐PH, either treated with solvent (top) or KI‐MIX (bottom) for 12 h, under baseline (ctrl) conditions, during perfusion of 10 μm OxoM and after washout (wash). *F*, The fluorescence signal relative to its starting signal (F/F_0_) of single cytosolic regions of interest before (ctrl) and during OxoM perfusion from solvent‐ (*n* = 7) or KI‐MIX‐ (*n* = 5) treated neurons were compared (Kruskal–Wallis, Dunn's *post hoc* multiple comparison).

The results showed that the KI‐MIX incubation greatly attenuated the maximal inhibition of M‐currents in SCG neurons by OxoM (solvent: 94.93 ± 1.97% *versus* KI‐MIX: 34.09 ± 4.19%; Fig. 6 *A* and *C*). In contrast to M_1_ mAChRs, B2 bradykinin receptors of SCG neurons mediate an inhibition of M‐currents not via PIP_2_ depletion, but through increases in intracellular Ca^2+^ (Delmas & Brown, 2005). When concentration–response curves for the inhibition of M‐currents by bradykinin were compared for KI‐MIX‐ and solvent‐treated neurons, no difference was seen (maximal inhibition solvent: 52.78 ± 7.46% *versus* KI‐MIX: 49.03 ± 4.49%; Fig. 6 *B* and *D*).

In order to determine whether the KI‐MIX affected M_1_ mAChR‐mediated PIP_2_ hydrolysis in SCG neurons, freshly dissociated SCG neurons were transfected with a YFP‐tagged pleckstrin homology domain of PLCδ (YFP‐PH). This binds specifically to PIP_2_ and thus enables the monitoring of PIP_2_ hydrolysis by its dissociation from the plasma membrane (Heo *et al*. 2006). Transfected neurons were incubated overnight in solvent or KI‐MIX and thereafter imaged using a confocal microscope.

The results showed that cytosolic fluorescence increased during stimulation with 10 μm OxoM, but did not differ between solvent and KI‐MIX‐treated cultures (Fig. 6 *E* and *F*). Thus, the effect of protein kinase inhibition on the regulation of M‐currents via GPCRs in sympathetic neurons appeared to be specific for the M_1_ mAChRs and occurred downstream of PIP_2_ depletion.

## Discussion

Kv7 channels represent a typical example of voltage‐gated ion channels that require PIP_2_ for opening. The function of these, and virtually all other, ion channels is additionally controlled by phosphorylation. Here, we addressed the link between these two key mechanisms by revealing a novel contribution of direct multisite phosphorylation within Kv7.2 to the channel's PIP_2_ sensitivity. Multiple constitutive phosphorylation sites were unambiguously identified in Kv7.2 by LC‐MS/MS. The detected phosphosites resided primarily in two clusters, one of which overlapped with a putative PIP_2_ binding domain. Five serine residues were found to be phosphorylated in this region that extended from residues K425 to K469 between helices A and B in the Kv7.2 C‐terminus (Hernandez *et al*. 2008). Interestingly, a previous mass spectrometry study proposed the potential phosphorylation of T217 in Kv7.2 (and T246 in Kv7.3) (Surti *et al*. 2005). This putative phosphorylation site is located in the S4–S5 linker close to the PIP_2_B binding domain (Fig. 1 *C*), where PIP_2_ is thought to interact with K230 of Kv7.2 channels (Zhang *et al*. 2013). Phosphomimetic mutations of T217 in Kv7.2 and T246 in Kv7.3 completely remove currents through these channels (Surti *et al*. 2005), either via dysregulated movement of the voltage sensor or altered PIP_2_ interaction. However, these phosphorylation sites have not been validated by MS/MS, either in the present work or in other large‐scale proteome studies (Trinidad *et al*. 2008, 2012; Tweedie‐Cullen *et al*. 2009; Huttlin *et al*. 2010; Wisniewski *et al*. 2010; Lundby *et al*. 2012). Additionally, it is not clear under which conditions these residues are phosphorylated or which kinase might be involved; however, the dephosphomimetic mutation of these sites did not affect channel inhibition via carbachol and caused a depolarizing shift in voltage‐dependent activation (Surti *et al*. 2005), which was not observed in our KI‐MIX‐treated Kv7.2 channels (Fig. 5 *D*). Hence, we may rule out an unspecific effect of KI‐MIX on this particular phosphorylation site.

When testing the sensitivities of wt or dephosphomimetic channels towards PIP_2_ by comparing time–effect curves for current reduction by Dr‐VSP activation, the apparent maximum inhibition in Kv7.3 was less than in Kv7.2 wt or its A^5^ mutant. Considering these relative values of current diminution, one should bear in mind the huge differences in current densities carried by homomeric Kv7.2 and Kv7.3 channels (Selyanko *et al*. 2001). Here, the densities of the currents passing through Kv7.3 were about fivefold less than those through Kv7.2 prior to Dr‐VSP activation, but there was no significant difference thereafter (Fig. 2 *C*). In contrast, the A^5^ mutant displayed a current density similar to that of wt Kv7.2 (Fig. 2 *J*). Taking this issue into account, we presume that the comparably reduced maximal effect of Dr‐VSP activation seen with Kv7.3 (Fig. 2 *A*) was probably related to the large differences in current densities rather than to differences in PIP_2_ sensitivities.

PIP_2_ is a negatively charged lipid thought to interact electrostatically with positively charged amino acids (Rosenhouse‐Dantsker & Logothetis, 2007). As the attachment of a phosphate group adds two negative charges to proteins, multisite phosphorylation in polybasic clusters of proteins may disturb an electrostatic interaction with PIP_2_ (Narayanan & Jacobson, 2009). Similarly, removal of negative charges may strengthen electrostatic interactions between proteins and PIP_2_. The higher the affinity of a channel for PIP_2_, the less sensitive the currents through that channel are towards PIP_2_ depletion (Gamper & Shapiro, 2007). Accordingly, currents through dephosphomimetic A^5^ mutants were less sensitive than wt channels towards Dr‐VSP activation (Fig. 2 *H*). Nevertheless, a lower expression level of Dr‐VSP may also cause slower deactivation of currents through Kv7.2 channels via Dr‐VSP activation. Dr‐VSP is a phosphoinositide phosphatase linked to a canonical voltage sensor and, hence, displays measurable ‘sensing’ currents that can be viewed as counterparts of gating currents in voltage‐gated ion channels (Okamura *et al*. 2009). Conversely, gating currents of Kv7.2 and Kv7.3 channels cannot be detected even after removing macroscopic currents by adding TEA to the extracellular solution (Miceli *et al*. 2009). Whenever possible, such ‘sensing’ currents of Dr‐VSP were recorded after the time‐dependent deactivation protocol. The OFF‐‘sensing’ charge movement from +140 mV to −60 mV was comparable for all groups investigated (Figs 2 *D* and *K*, and 5 *C*, *I*, *L* and *O*). Thus, we attributed the observed slower deactivation of the dephosphomimetic mutant to the channel's PIP_2_ sensitivity and not to variability in Dr‐VSP expression.

Recently, Dr‐VSP was found to not only remove the phosphate at position 5 of the inositol ring of PI(4,5)P_2_, but also that of PI(3,4,5)P_3_, as well as the phosphate at position 3 of PI(3,4)P_2_ and PI(3,4,5)P_3_. Together these reactions lead to the formation of PI(4)P via one or two steps. However, PI(4,5)P_2_ is the most abundant phosphoinositide in the membrane and the phosphatase action at position 5 of PI(4,5)P_2_ occurs at a much higher rate than the other reactions, therefore resulting in the predominant Dr‐VSP activity (Keum *et al*. 2016). Heteromeric Kv7.2/7.3 channels are activated by PI(3,4,5)P_3_ as well as by PI(4,5)P_2_ with similar potency. Furthermore, PI(4)P is also able to activate these channels with a slightly lower potency (Zhang *et al*. 2003; Telezhkin *et al*. 2012). Nevertheless, activation of Dr‐VSP completely abolishes currents through Kv7 channels in a time‐ and voltage‐dependent manner (Falkenburger *et al*. 2010), thus obviously resulting in a sufficiently diminished phosphoinositide content of the membrane. Yet, intermediate products of phosphoinositide dephosphorylation may substitute for PI(4,5)P_2_ during Dr‐VSP activity and hence one cannot directly compare apparent PIP_2_ affinities of various Kv7 subunits with results from VSP experiments.

*In vitro* protein kinase assays on recombinant Kv7.2 C‐termini followed by MS identified CDK5, p38 MAPK, CaMKIIα and PKA as specific protein kinases responsible for the phosphotransfer to the five phosphoserines contained within the cationic PIP_2_ binding region (Table 2). Inhibitors of these four protein kinases lessened the *in vitro* phosphorylation within PIP_2_D (Fig. 4 *A*) and rendered currents through Kv7.2 channels less sensitive towards PIP_2_ depletion (Fig. 5 *A*). However, we cannot exclude the involvement of other protein kinases due to the limited number of applied protein kinases *in vitro* and non‐selectivity of inhibitors: the pan‐CDK‐inhibitor roscovitine (Meijer *et al*. 1997; Bain *et al*. 2007); SB203580 inhibiting p38 MAPK, GSK3β and CK1δ amongst others (Bain *et al*. 2007); the CaMKII and GSK3β inhibitor KN‐62 (Davies *et al*. 2000); and H7, an inhibitor of PKA, PKC and PKG (Hidaka *et al*. 1984). Nevertheless, the curtailment of currents through Kv7.2 A^5^ mutants, Kv7.2 D^5^ mutants or Kv7.3 channels by Dr‐VSP activation was not altered by these protein kinase inhibitors (Fig. 5 *G*, *J* and *M*). These results indicate that channel phosphorylation determines the sensitivity of Kv7.2 for PIP_2_ and rule out untargeted effects of the KI‐MIX. Interestingly, Kv7.3 channels lack 4 out of 5 serines within their PIP_2_D domain (helix A–B linker site in Fig. 3 of Zaydman & Cui, 2014). It remains to be determined whether subunit‐specific phosphorylation of PIP_2_D might be relevant for the differences in PIP_2_ sensitivity.

Recently, phosphorylation of CaM has been reported to stabilize K^+^ channel–PIP_2_ interactions (Kang *et al*. 2014; Zhang *et al*. 2014). In SK channels, this decreases PIP_2_ affinity (Zhang *et al*. 2014), whereas in Kv7.2 channels this causes an increase (Kang *et al*. 2014). Even though the focus of this study was direct phosphorylation of a PIP_2_ domain in Kv7.2, potential concomitant effects on CaM need to be considered. However, the protein kinase that reportedly phosphorylates CaM tethered to Kv7.2 was CK2 (Kang *et al*. 2014), and none of the protein kinase inhibitors employed here is known to suppress CK2 activity. Even if the KI‐MIX might suppress CK2 and thus dephosphorylate CaM, one would expect a weaker affinity of PIP_2_, namely a higher sensitivity towards PIP_2_ depletion for Kv7.2. However, the opposite was true since an apparent decrease in PIP_2_ sensitivity of Kv7.2 was the outcome of KI‐MIX treatment. Therefore, we can exclude a contribution of CaM phosphorylation to the current data.

Under physiological conditions, PIP_2_ depletion occurs downstream of the activation of Gq‐coupled receptors, and has been documented to be the relevant mechanism underlying the inhibition of M‐currents in sympathetic neurons, via M_1_ receptors (Suh & Hille, 2002; Delmas & Brown, 2005). Treatment of SCG neurons with the protein kinase inhibitors that rendered recombinant Kv7.2, but not Kv7.3, channels less sensitive towards PIP_2_ depletion (Fig. 5 *A* and *M*) greatly reduced the maximal inhibition of M‐currents by OxoM (Fig. 6 *C*). In a heterologous expression system, a similar difference in maximal M_1_ receptor‐mediated inhibition of currents was observed between Kv7.3 homomers and Kv7.2/7.3 heteromers (compare present Fig. 6 *C* with Fig. 2 *B* of Hernandez *et al*. 2009). For the recombinant channels, this difference in M_1_ receptor‐mediated inhibition was related to the varying PIP_2_ affinities of Kv7.3 homomers and Kv7.2/7.3 heteromers (Hernandez *et al*. 2009) by a factor of 10 (Li *et al*. 2005; Hernandez *et al*. 2009; Telezhkin *et al*. 2012). The difference in current sensitivity towards Dr‐VSP activation was twofold for Kv7.2 *versus* Kv7.3 and similar for wt Kv7.2 *versus* the A^5^ mutant. Hence, it is tempting to speculate that dephosphorylation of the five serine residues in the interhelical PIP_2_ domain might lead to a similar increase in the apparent PIP_2_ affinity of Kv7.2.

With reference to the bradykinin‐mediated inhibition of Kv7 channels, this is independent of PIP_2_ depletion and instead involves rises in intracellular Ca^2+^ and CaM (Delmas & Brown, 2005). Accordingly, the KI‐MIX treatment here did not affect M‐current inhibition in SCG neurons by bradykinin (Fig. 6 *D*). This raised the question of whether Kv7.2 channel inhibition via either PIP_2_ depletion or Ca^2+^/CaM activation occur independently of each other. M_1_ receptor activation has been shown previously to activate PKC tethered to Kv7 channels via AKAP150, which leads to phosphorylation of rat Kv7.2 at S541 (Hoshi *et al*. 2003). S541 is close to the CaM binding motif (Fig. 1 *C*) and its phosphorylation causes dissociation of CaM from Kv7.2, thereby decreasing its PIP_2_ affinity (Kosenko *et al*. 2012). Of the protein kinase inhibitors employed here, H7 is known to also interfere with PKC; However, H7 impedes PKC activity via its catalytic site (Hidaka *et al*. 1984), and other blockers of PKC's catalytic site do not affect Kv7 inhibition via M_1_ receptors in SCG neurons (Hoshi *et al*. 2003). Hence, the effects of our KI‐MIX treatment were related to the inhibition of CDK5, CaMKIIα, p38 MAPK and PKA (Fig. 4 *A*) and independent of the well‐known role of PKC in Kv7 channel regulation (Hoshi *et al*. 2003; Nakajo & Kubo, 2005; Kosenko *et al*. 2012).

A reduction of sensitivity towards PIP_2_ depletion via Dr‐VSP activation was also previously seen in a deletion mutant of Kv7.2, where a part of the A–B linker region including the PIP_2_D was removed (Aivar *et al*. 2012). Yet, the authors could not find a statistical significance due to the high variability of their data. Since Kv7.2 channels are still functional and have a sufficient PIP_2_ interaction even without the A–B linker region (Aivar *et al*. 2012), PIP_2_D is not required for the PIP_2_ interaction, but rather appears to be a regulator of PIP_2_ sensitivity. In fact, PIP_2_D is the only PIP_2_‐binding domain showing variable sequences between the different Kv7 subunits, whereas the other three PIP_2_‐binding domains are well conserved (Zaydman & Cui, 2014).

In summary, the results presented here showed that the PIP_2_ sensitivity of Kv7.2 channels was directly affected by post‐translational modification. Phosphorylation of a protein as a reversible process usually acts like an on/off switch at a single phosphorylation site (Landry *et al*. 2014). In contrast, the multiple phosphorylation sites in Kv7.2 channels, some of which were phosphorylated by more than one protein kinase, hinted towards an orchestrated contribution to channel function by several phosphoresidues that were concentrated within a functional motif. It is tempting to speculate that the constitutive phosphorylation of Kv7.2 channels in the proximal cluster is needed to maintain the channel's low sensitivity towards PIP_2_ so as to enable tight regulation of the channel via Gq‐coupled receptors.

## Additional information

### Competing interests

None declared.

### Author contributions

I.S., F.A.E., S.B. and J.‐W.Y. designed the research; I.S., F.A.E., W.‐Q.C., S.H., X.K., K.W.S. and J.‐W.Y. performed research; H.K. and G.L. contributed new reagents/analytical tools; I.S., F.A.E., W.‐Q.C., S.H., K.W.S., S.B., and J‐.W.Y. analysed data; I.S., F.A.E., S.B. and J.‐W.Y. wrote the manuscript. All authors have approved the final version of the manuscript and agree to be accountable for all aspects of the work. All persons designated as authors qualify for authorship, and all those who qualify for authorship are listed.

### Funding

This work was supported by the Austrian Science Fund (FWF): W1205 (to S.B.), P19710 (to H.K.) and P23670 (to J.‐W.Y.). I.S. is a member of the doctoral programme ‘Cell Communication in Health and Disease’ (CCHD; co‐financed by FWF and the Medical University of Vienna).
